# Supplementary material for: Comparative analysis of deep learning algorithms for dental caries detection and prediction from radiographic images: a comprehensive umbrella review
Source: PeerJ Comput Sci. 2024 Nov 12;10:e2371. doi: 10.7717/peerj-cs.2371 (PMC11622875; doi:10.7717/peerj-cs.2371)
Supplement: Supplemental Information 7 [file peerj-cs-10-2371-s007.docx]

**The rationale for conducting the systematic review**

Untreated dental caries can lead to severe oral disease, emphasizing that early diagnosis and treatment is crucial. Fortunately, recent advancements in AI and machine learning - such as deep learning and neural networks - have shown promise in various medical applications, including dental radiography. We can significantly improve patient outcomes and the treatment prognosis by utilizing AI to increase the speed and accuracy of dental caries diagnosis. Our systematic review evaluates the accuracy of these algorithms in detecting and predicting dental caries through radiographical images. By providing valuable insights into this field, we aim to help healthcare professionals understand the potential of AI for detecting dental caries and its limitations.

**The contribution that it makes to knowledge in light of previously published related reports, including other meta-analyses and systematic reviews.**

The field of AI-powered dental caries detection lacks a comprehensive and overview over multiple systematic reviews published recently. The aim of the present Umbrella review is to fill this gap by providing an extensive overview of published systematic reviews and a critical appraisal of their findings. By assessing the quality of these reviews and referencing the most frequently cited original works. The present Umbrella review aims to offer a more nuanced and comprehensive understanding of the limitations and achievements of AI technology in the realm of dental caries detection. This review will be of great help to researchers, practitioners and scientists in making informed decisions and developing effective strategies for future research.
