# Supplementary material for: Comparative analysis of deep learning algorithms for dental caries detection and prediction from radiographic images: a comprehensive umbrella review
Source: PeerJ Comput Sci. 2024 Nov 12;10:e2371. doi: 10.7717/peerj-cs.2371 (PMC11622875; doi:10.7717/peerj-cs.2371)
Supplement: Supplemental Information 8 [file peerj-cs-10-2371-s008.docx]

The Joanna Briggs Institute (JBI) checklist questions for quality assessments of systematic reviews.

| **Domain number** | **Question** |
| --- | --- |
| 1 | Is the review question clearly and explicitly stated? |
| 2 | Were the inclusion criteria appropriate for the review question? |
| 3 | Was the search strategy appropriate? |
| 4 | Were the sources and resources used to search for studies adequate? |
| 5 | Were the criteria for appraising studies appropriate? |
| 6 | Was critical appraisal conducted by two or more reviewers independently? |
| 7 | Were there methods to minimize errors in data extraction? |
| 8 | Were the methods used to combine studies appropriate? |
| 9 | Was the likelihood of publication bias assessed? |
| 10 | Were recommendations for policy and/or practice supported by the reported data? |
| 11 | Were the specific directives for new research appropriate? |
